# Functional Expression and Characterization of Acetyl Xylan Esterases CE Family 7 from *Lactobacillus antri* and *Bacillus halodurans*

**DOI:** 10.4014/jmb.2001.01004

**Published:** 2020-01-17

**Authors:** Min-Jeong Kim, Myoung-Uoon Jang, Gyeong-Hwa Nam, Heeji Shin, Jeong-Rok Song, Tae-Jip Kim

**Affiliations:** Division of Animal, Horticultural and Food Sciences, Graduate School of Chungbuk National University, Cheongju 28644, Republic of Korea

**Keywords:** Acetyl xylan esterases, carbohydrate esterase (CE) family 7, synergistic xylan degradation, *Lactobacillus antri*, *Bacillus halodurans*

## Abstract

Acetyl xylan esterase (AXE; E.C. 3.1.1.72) is one of the accessory enzymes for xylan degradation, which can remove the terminal acetate residues from xylan polymers. In this study, two genes encoding putative AXEs (LaAXE and BhAXE) were cloned from *Lactobacillus antri* DSM 16041 and *Bacillus halodurans* C-125, and constitutively expressed in *Escherichia coli*. They possess considerable activities towards various substrates such as *p*-nitrophenyl acetate, 4-methylumbelliferyl acetate, glucose pentaacetate, and 7-amino cephalosporanic acid. LaAXE and BhAXE showed the highest activities at pH 7.0 and 8.0 at 50°C, respectively. These enzymes are AXE members of carbohydrate esterase (CE) family 7 with the cephalosporine-C deacetylase activity for the production of antibiotics precursors. The simultaneous treatment of LaAXE with *Thermotoga neapolitana* β-xylanase showed 1.44-fold higher synergistic degradation of beechwood xylan than the single treatment of xylanase, whereas BhAXE showed no significant synergism. It was suggested that LaAXE can deacetylate beechwood xylan and enhance the successive accessibility of xylanase towards the resulting substrates. The novel LaAXE originated from a lactic acid bacterium will be utilized for the enzymatic production of D-xylose and xylooligosaccharides.

## Introduction

Xylans are one of the most abundant hemicellulosic bioresources and are widely distributed in plants. These β-(1,4)-linked D-xylanopyranosyl polymers possess various side chains being substituted with L-arabinose, α-glucuronic acid, acetate, and ferulic acid residues. Therefore, the complete xylan degradation requires the cooperative actions of several accessory enzymes with the major xylan hydrolases such as *endo*-β-xylanases [1-4].

Acetyl xylan esterase (AXE, E.C. 3.1.1.72) is the xylanolytic accessory enzyme hydrolyzing the ester linkages of xylans and/or acetylated sugars to remove the acetate residues. The deacetylating activity of AXE can facilitate the hydrolytic actions of β-xylanase to produce higher amounts of D-xylose and xylooligosaccharides (XOSs) from agricultural wastes [4, 5]. The resulting products are utilized as functional sugar substitutes for diabetics and as fermentable sugars for bioethanol production [4, 6].

In the carbohydrate active enzyme database (CAZy; http://www.cazy.org), AXEs are categorized to the carbohydrate esterase (CE) families 1~7, 12, and 16, on the basis of their amino acid sequence similarity, substrate specificity, and protein structure [5]. A number of AXEs have been studied from various bacteria including *Bacillus*, *Thermoanaerobacterium*, *Streptomyces*, *Aspergillus*, *Trichoderma*, and *Thermotoga* species [7, 8]. The majority of AXEs are the serine-type esterases with activity on small substrates such as 4-MUAc and *p*-NPAc. Among them, some microbial AXEs with cephalosporin-C deacetylase (CCD; E.C. 3.1.1.41) activity have been categorized to CE family 7 [5]. The microbial AXEs CE7 have been reported from *B. subtilis* SHS0133 [9], *B. pumilus* PS213 [10], *Thermotoga maritima* [11], *B. subtilis* CICC20034 [12], and so on. These enzymes could produce β-lactam antibiotics by using the deacetylated cephalosporin or 7-aminocephalosporanic acid (7-ACA) as the intermediates [13, 14].

Similar to the approved prebiotics of fructooligosaccharides (FOSs) and galactooligosaccharides (GOSs), XOSs with a degree of polymerization of 2~10 are considered as prebiotic candidates promoting the growth of probiotics, *Bifidobacterium* [15] and *Lactobacillus* species [16]. The health-beneficial effects of XOSs are a wide range of biological functions including antioxidant, antimicrobial, antiallergy, antiinfection, anti-inflammatory, and immuno-modulatory activities [6]. Nevertheless, only a few studies about the synergistic effects of AXEs and β-xylanases have been done to date.

In the present study, a putative gene encoding AXE CE7 has been firstly expressed and characterized from *Lactobacillus antri*, a lactic acid bacterium. As a reference model enzyme, an AXE CE7 from *Bacillus halodurans* was also cloned and functionally expressed in *E. coli*. Especially, their CCD activities and synergistic effects on beechwood xylan degradation with β-xylanase were comparatively characterized with the other known microbial AXEs.

## Materials and Methods

### Enzymes and Reagents

A DNeasy Blood/Tissue Kit (Qiagen, Germany) was used for the preparation of bacterial genomic DNA. Oligonucleotide primers, an *Accuprep* Plasmid Extraction Kit and a PCR Purification Kit were supplied by Bioneer (Korea). The restriction endonucleases and *Pyrobest* DNA polymerase were purchased from Takara Biomedical (Japan). Various substrates of *p*-nitrophenyl acetate (*p*-NPAc), 4-methylumbelliferyl acetate (4-MUAc), β-D-glucose pentaacetate (GPAc), 7-aminocephalosporanic acid (7-ACA), and beechwood xylan (BEX) were obtained from Sigma-Aldrich (USA).

### Gene Cloning of AXEs and XN

The genomic DNA templates were prepared from the cultures of *Lactobacillus antri* DSM 16041 (=KCTC 5855) and *Bacillus halodurans* C-125 grown in nutrient broth (0.5% peptone, 0.3%meat extract, and 1.0% MnSO_4_) at 30°C and in Luria-Bertani (LB) broth (1.0% tryptone, 1.0% sodium chloride, and 0.5% yeast extract) at 37°C, respectively. The sets of PCR primers, LaAXE-N (5’-TTTTCATATGCAAGATATTCAGCAAT-3’), LaAXE-C (5’-TTTTCTCGAGGTCTTTGCCAATTTGAAT-3’), BhAXE-N (5’-TTTTCATATGCCACTAATAGACATG-3’), and BhAXE-C (5’-TTTTCTCGAGGAGATCAGATAAAAATTGAA-3’) were used for the gene amplification of LaAXE (GenBank ID: EEW54661.1) and BhAXE (BAB07045.1), respectively. PCR was carried out using a C-1000 thermal cycler (Bio-Rad, Hercules, USA) for 30 cycles at 98°C for 30 sec, 50~52°C for 30 sec, and 72°C for 60 sec. The PCR-amplified fragment was digested with NdeI and XhoI, and cloned into an expression vector, pHCXHD [17]. The resulting recombinant plasmids were designated as pHCLaAXE and pHCBhAXE, respectively. For the gene cloning of β-xylanase (TnXNB; AAN16480.1) from *Thermotoga neapolitana* DSM 4359, the corresponding gene was amplified by using a pair of PCR primers, TnXNB-N (5’-TTTTGAATTCATGAAAGGGTTGCCTGC-3’) and TnXNB-C (5’-TTTTCTCGAGTCTTTCCTTCAGTTTTTCCT-3’). The amplified fragment digested with EcoRI and XhoI was cloned into pHCXHD, which was designated as pHCTnXNB.

### Gene Expression and Enzyme Purification

Each recombinant *E. coli* MC1061 harboring pHCLaAXE, pHCBhAXE, or pHCTnXNB was grown in LB broth containing 100 μg/ml of ampicillin at 37°C for 12 h. *E. coli* cells were disrupted by ultrasonicator VCX750 (Sonics & Materials, USA). The recombinant enzyme with C-terminal six-histidines was purified by using Ni-NTA affinity chromatography (Qiagen). The purity and the molecular mass of each enzyme were determined by using 12% SDS-PAGE analyses. The protein concentration was measured using the BCA Protein Assay Kit (Pierce Biotechnology, USA) with bovine serum albumin as a standard.

### AXE Activity Assays

For the activity assay on *p*-NPAc, 1 mM of *p*-NPAc was reacted with AXE and the absorbance of p-nitrophenol released was measured at 405 nm. For the activity assay on 4-MUAc, the resulting absorbance was measured at 354 nm from 0.4 mM of substrate. One unit of AXE activity on *p*-NPAc or 4-MUAc was defined as the enzyme amount releasing 1 μmol of each hydrolyzed product per min. In order to determine the AXE activity towards GPAc or 7-ACA, each AXE was reacted with 20 mM of each substrate. The resulting acetate concentration was measured by high-performance liquid chromatography (HPLC) Acme 9000 (Young-Lin, Korea) with UV detector at 210 nm, Aminex HPX-87H column (300 mm × 7.8 mm, Bio-Rad), and 0.6 ml/min of 0.008 N sulfuric acid as a mobile phase. One unit of AXE activity on each substrate was defined as the enzyme amount for liberating 1 μmol of acetate equivalent per min.

### Xylanase Activity Assay and Synergistic Effect Analysis 

The 3,5-dinitrosalicylic acid (DNS) reducing sugar assay was used to determine the TnXNB activity on 2.5% of BEX, and the absorbance of reducing sugar was measured at 550 nm. One unit of enzyme activity was defined as the TnXNB amount for producing 1 μmol of reducing sugar as D-xylose equivalent per min. In order to examine the synergistic effects of the simultaneous treatment with TnXNB and AXE, 2.5% of BEX was reacted with TnXNB (8 mU/ml on BEX) and each AXE (2 U/ml on 4-MUAc) under the controlled reaction condition. For the stepwise enzyme treatment, each AXE was reacted with 2.5% of BEX for 4 h and then inactivated by boiling for 5 min. The resulting reaction mixture was further treated with TnXNB for an additional 4 h. The amount of reducing sugar liberated was determined by DNS assay. LaAXE and BhAXE were reacted with BEX in 50 mM sodium acetate (pH 7.0) at 60°C and in 50 mM Tris-HCl (pH 7.0) at 50°C, respectively.

### Thin-Layer Chromatography Analysis

Thin-layer chromatography (TLC) was employed for the time-course analysis of GPAc hydrolysis. 0.1 U/ml of each AXE was reacted with 50 mM of GPAc under its optimal conditions for each time period. The resulting hydrolysis products were separated on a 60F254 silica gel plate (Merck) using the solvent mixture of ethylacetate:benzene:isopropanol (2.0:1.0:0.1). The spots were detected by dipping the plate in the developing solution (0.3% N-1-naphthyl-ethylenediamine and 5% H_2_SO_4_ in methanol), and then heating it at 110°C for 10 min.

## Results and Discussion

### Gene Cloning and Expression of AXEs

It was reported that a marine isolate, *Lactobacillus* sp., showed xylanolytic enzyme activities such as α-L-arabinofuranosidase and AXE [18]. However, the related genes and enzymes have not been characterized from lactic acid bacteria yet. In the present study, the gene encoding a putative AXE (LaAXE) has been found for the first time from the genome of *Lactobacillus antri* DSM 16041 [19]. The structural gene of LaAXE (GenBank ID: EEW54661.1) consisting of 972 bp encoding 323 amino acids (37 kDa) was PCR-amplified and cloned into an expression vector of pHCXHD, which was designated as pHCLaAXE (4,695 bp). LaAXE showed below 38% of amino acid sequence identities with the other known bacterial AXEs [7, 8]. As a reference AXE enzyme, the other putative gene encoding BhAXE (BAB07045.1; 319 amino acids and 36 kDa) was also cloned from an alkaliphile, *B. halodurans* C-125 [20]. BhAXE shares the highest amino acid sequence identity (37.8%) with LaAXE. Most of the AXEs from *Bacillus* spp. were known to possess not only AXE activity, but also CCD activity. On the contrary, LaAXE showed much lower sequence identities (below 15%) to the AXEs from *Trichoderma reesi* [21] and *Aspergillus oryzae* [22]. These fungal AXEs were reported to possess only AXE activity without CCD activity.

**Fig. 1:**
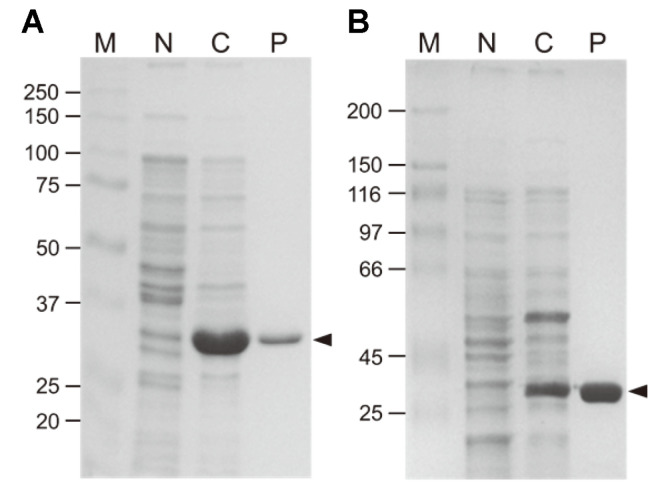
SDS-PAGE analysis for gene expression and enzyme purification of LaAXE (**A**) and BhAXE (**B**) from recombinant *E. coli*. Lanes M, protein size marker; N, cell extract from *E. coli* harboring an empty vector (pHCXHD) as a negative control; C, cell extract from *E. coli* containing AXE gene; P, recombinant AXE purified by Ni-NTA chromatography. The AXE bands are indicated by arrowheads.

**Fig. 2:**
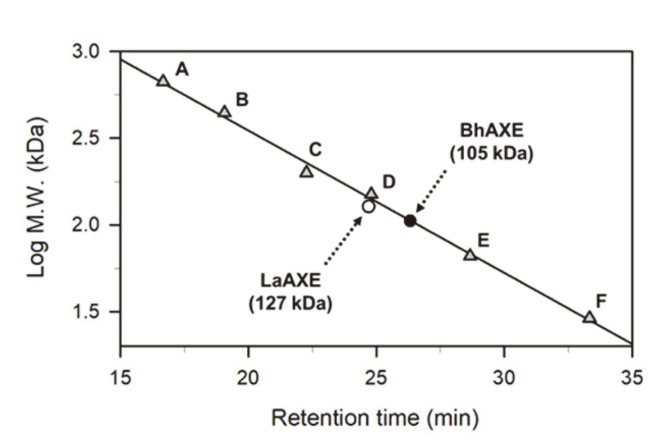
Quaternary structure estimation of AXEs by gel permeation chromatography with Superdex 200 column. The molecular weights of LaAXE and BhAXE were indicated by open and closed circles, respectively. Protein molecular weight standards (triangles) consist of thyroglobulin (**A**, 669 kDa), Apoferritin (**B**, 443 kDa), β-amylase (**C**, 200 kDa), alcohol dehydrogenase (**D**, 150 kDa), bovine serum albumin (**E**, 66 kDa), and carbonic anhydrase (**F**, 29 kDa).

The recombinant LaAXE or BhAXE with C-terminal six-histidines was constitutively expressed and purified from *E. coli* harboring pHCLaAXE or pHCBhAXE via Ni-NTA affinity chromatography (Fig. 1). The gel permeation chromatography analyses showed that the native LaAXE and BhAXE are likely to exist as the homo-trimeric or tetrameric forms (apparent molecular mass of 127 and 105 kDa, respectively) in solution (Fig. 2). It was revealed that the representative AXE CE family 7 from *B. pumilus* has the doughnut-like hexameric conformation [23]. Another highly thermostable AXE CE7 from *T. maritima* was a homo-hexamer in solution, but a dimeric form was also observed [24].

### Enzymatic Characterization of AXEs

**Fig. 3:**
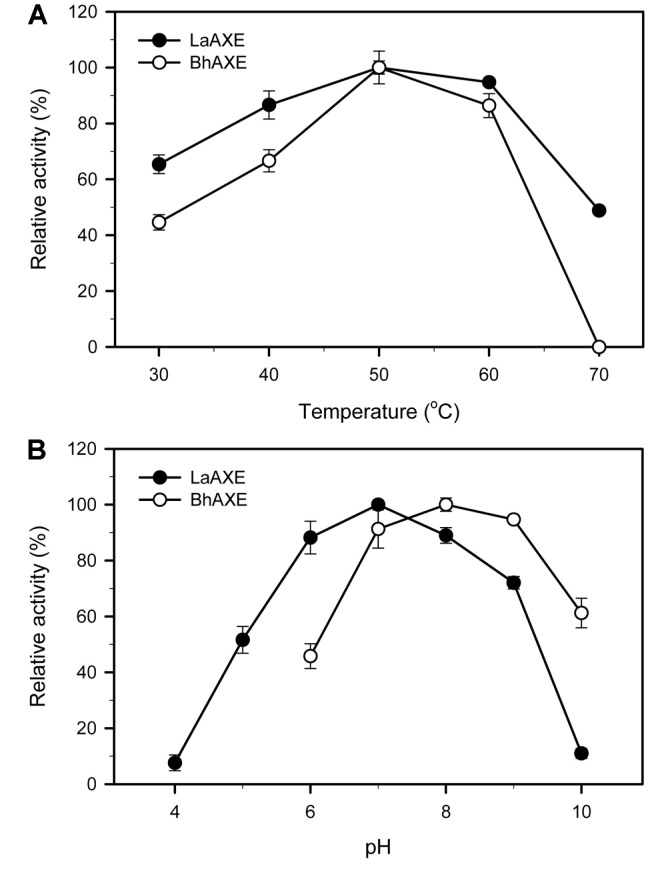
Effects of temperature (**A**) and pH (**B**) on the activities of LaAXE and BhAXE. Relative activities of AXEs on glucose pentaacetate (GPAc) were determined at different temperature and pH conditions.

Due to the low stability of 4-MUAc at the alkaline pH above 9.0, the optimal reaction conditions of LaAXE and BhAXE were determined by using HPLC analysis with GPAc as a substrate. Both AXEs showed the highest activities at 50°C in 50 mM sodium phosphate buffer at pH 7.0 (Fig. 3A). At the high temperature of 70°C, the activity of LaAXE decreased to 48.8%, whereas BhAXE lost almost all its activity. LaAXE and BhAXE showed the highest activities in 50 mM sodium phosphate buffer (pH 7.0) and Tris-HCl buffer (pH 8.0), respectively (Fig. 3B). While LaAXE was stable at the broad pH ranges between 6.0 and 11.0, only less than 30% of its activity remained at the pH ranges below 5.0 or above 12.0. BhAXE showed high stability at the pH ranges between 7.0 and 10.0 (data not shown). The common microbial AXEs possess their optimal reaction conditions at the pH ranges of 6.0~8.0 and at temperatures from 30 to 70°C [7, 8]. The highly thermostable AXEs were reported from the thermophiles such as *T. maritima* [24], *Thermoanaerobacterium* sp. [25], and *Thermobifida fusca* [26]. The fungal AXEs from *Aspergillus oryzae* [22] and *Trichoderma reesei* [21] are the mesophilic enzymes that are highly active at 45~65°C, whereas a cold-active AXE was also reported from *Paenibacillus* sp. [27].

### Substrate Specificities of AXEs

In order to determine the substrate specificity of AXEs, the enzyme reactions were conducted at its optimal reaction conditions with various substrates including GPAc, 4-MUAc, *p*-NPAc, and 7-ACA. As shown in Table 1, LaAXE showed the highest specific activity of 499.5 U/mg on GPAc. Among the synthetic substrates, *p*-NPAc is the much better substrate for LaAXE than 4-MUAc. Expectedly, it was found that LaAXE possesses the detectable deacetylating activity on 7-ACA (20.7 U/mg), even though this activity is only 4.1% of that on GPAc. Meanwhile, BhAXE showed the highest specific activity of 501.0 U/mg on GPAc. Especially, it has 8.0 times higher activity on 7-ACA (165.0 U/mg) than LaAXE, which corresponds to 32.9% of the activity towards GPAc.

In general, the hemicellulose-deacetylating AXEs are members of CE families 1~7 [8]. The substrate preferences of AXEs are highly dependent on the microbial origins. For example, the AXE from *Bacillus subtilis* CICC20034 showed the highest activity (2,949.0 U/mg) on p-NPAC, whereas its relative activities on GPAc and 4-MUAc are 42.2 and 36.8%of that on *p*-NPAc, respectively [12]. On the other hand, GPAc is the most preferred substrate for the AXE from *B. pumilus* PS213 [10].

As LaAXE and BhAXE showed the considerable deacetylating activities on 7-ACA, these enzymes could be categorized into the AXEs CE family 7. The cephalosporin-C deacetylases (CCDs) are the carbohydrate esterases which hydrolyze the ester linkages of the acetyl groups of 7-ACA as well as the acetylated xylans [5, 12]. BhAXE showed the specific activity of 165.0 U/mg on 7-ACA, which is similar with that of CCD from *Bacillus pumilus* PS213 [10].

**Fig. 4:**
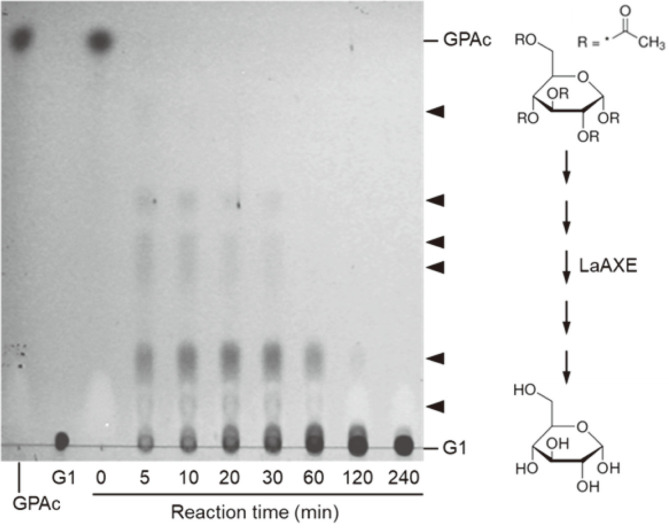
Time-course TLC analysis of glucose pentaacetate (GPAc) deacetylation catalyzed by LaAXE. 0.1 U/ml of enzyme was reacted with 50 mM GPAc in 50 mM sodium phosphate buffer (pH 7.0) at 50°C. The putative deacetylated intermediates from GPAc to glucose (G1) are indicated by the arrowheads.

GPAc contains a total of five acetate groups linked to C-1, C-2, C-3, C-4, and C-6 of glucose. The time-course study was performed to investigate the deacetylation patterns of GPAc by AXEs. When LaAXE was reacted with GPAc, a series of deacetylated intermediates were detected by TLC analysis (Fig. 4). BhAXE showed almost identical deacetylation patterns of GPAc to LaAXE. Although the exact chemical structures of each intermediate compound were not identified, LaAXE and BhAXE are likely to catalyze the stepwise deacetylation of GPAc to produce only glucose as an end-product. Their detailed positional specificity AXE should be further investigated.

### Synergistic Effect on Xylan Degradation

**Fig. 5:**
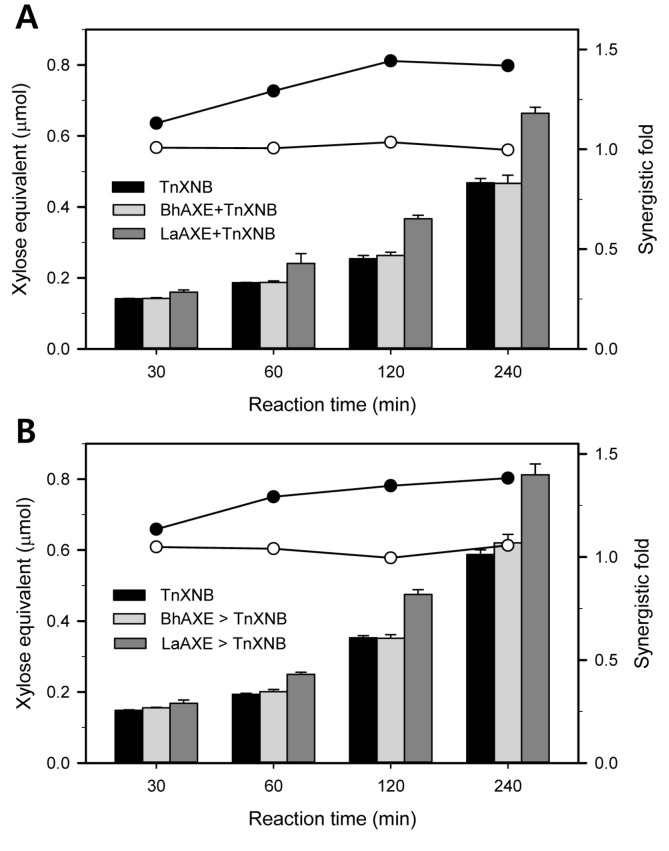
Synergistic degradation of beechwood xylan (BEX) via the simultaneous (**A**) and the stepwise (**B**) treatments of AXE and TnXNB. TnXNB (8 mU/ml) and each AXE (2.0 U/ml) were reacted on 2.5% of BEX in 50 mM sodium acetate (pH 5.5) at 60 °C with LaAXE and Tris- HCl (pH 7.0) at 50°C with BhAXE for 4 h, respectively. The xylose equivalent was measured by DNS reducing sugar assay. The synergistic folds were shown as the solid lines with the symbols of closed (LaAXE) and open (BhAXE) circles, respectively.

AXE can remove the acetate groups from the heteroxylans, which facilitates the successive actions of main xylan-hydrolases such as β-xylanase and β-xylosidase [4]. The synergistic effects of AXEs on the xylan degradation were investigated with β-xylanase. β-Xylanase from *Thermotoga neapolitana* DSM 4359 (TnXNB, GenBank ID: AAN16480.1) is one of the typical *endo*-hydrolases, which efficiently hydrolyzes beechwood xylan (BEX) to produce xylobiose and xylotriose as the major products [28]. The simultaneous treatment of LaAXE and TnXNB showed the maximal synergistic fold of 1.44 for 2 h (Fig. 5A). The stepwise treatment of LaAXE to TnXNB also showed the highest synergistic fold of 1.38 for 4 h (Fig. 5B). Although the simultaneous treatment showed slightly higher synergistic fold than the stepwise treatment, the difference between them seemed negligible. These results implied that LaAXE could attack both oligomeric and polymeric BEX to generate the deacetylated substrates. TnXNB could easily hydrolyze the resulting deacetylated substrates to shorter products. In case of *Volvariella volvacea*, the stepwise treatment of AXE to XN showed the higher synergistic fold of 1.4 than the simultaneous treatment of 1.1-fold [2]. The simultaneous treatments of AXE and XN from *Streptomyces* sp. and *Thermobifida fusca* on oat-spelt xylan showed the highest synergistic folds of 1.3 [1]. The AXE from *Ochrovirga pacifica* showed 1.4-fold of synergism on BEX with a commercial xylanase [3].

Unexpectedly, the maximal synergistic folds of BhAXE and TnXNB were 1.03 and 1.06 by the simultaneous and the stepwise treatments, respectively (Fig. 5). Even though the reasons for these differences between them have not been unveiled yet, the modes of action of each AXE were obviously distinguished from the other. The reasons why BhAXE cannot make significant synergistic effects on BEX degradation should be further investigated with the different types of xylans and β-xylanases. These considerable synergistic actions of LaAXE can be utilized for the efficient degradation of xylan polymers to produce the functional carbohydrates, D-xylose and/or XOSs.

### Enzyme Classification of AXEs

Various types of semi-synthetic β-lactam antibiotics, such as cefoxitin and cefuroxime, require the deacetylation of cephalosporin-C or 7-ACA as the precursor compounds [13]. To date, the AXE CE7 from *B. subtilis* CICC 20034 with the highest deacetylating activity on 7-ACA (888 U/mg) was developed for the large-scale enzymatic production of the deacetylated 7-ACA [12]. The improved activity and specificity of AXE CE7 or CCD should be needed for the efficient industrial production of deacetyl cephalosporin-C and 7-ACA [12, 14].

**Fig. 6:**
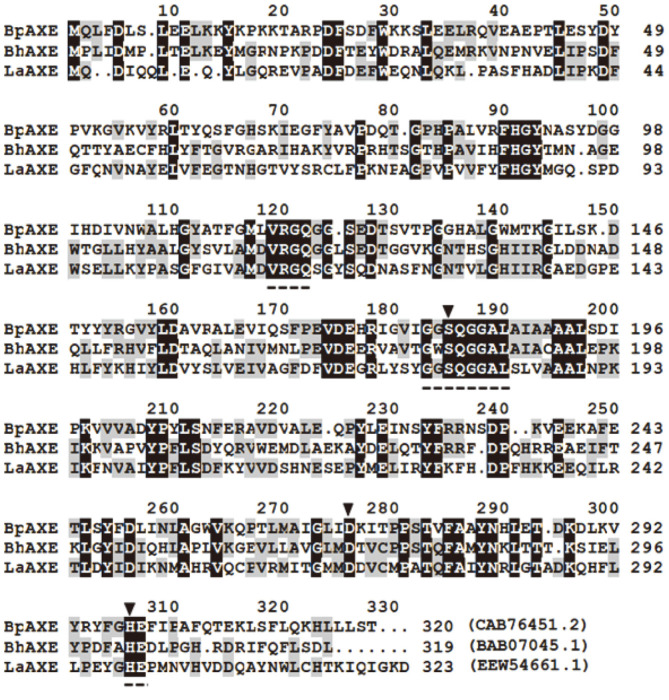
Amino acid sequence alignment among bacterial AXEs CE family 7. The fully conserved amino acid residues were shaded in black and the catalytic triad (Ser-Asp-His) is indicated by arrowheads. The conserved residues related to CCD activity are shown by dashed underlines. BpAXE from *Bacillus pumilus*; BhAXE from *B. halodurans*; LaAXE from *Lactobacillus antri*.

The genes encoding AXEs and the CCDs may be divergently evolved from a common ancestor belonging to the CE family 7. The phylogenetic analyses revealed that both LaAXE and BhAXE are closely related to the AXEs CE7 originated from *Bacillus* species, whereas they are distantly located from the fungal AXEs without the CCD activity. The amino acid sequence alignment revealed that three separate amino acid sequence motifs (RGQ, GxSQGG, and HE) are highly conserved in LaAXE, BhAXE, and the other known AXE CE7 from *Bacillus pumilus* [23] (Fig. 6). These three conserved motifs with the appropriate spacing have been defined as the signature features of CE family 7 enzymes [29]. In addition, the putative catalytic triad residues (Ser178, Asp268, and His298 in LaAXE) were well-conserved in these three AXEs, which were commonly found from the AXEs CE7 with the CCD activity.

Based on the amino acid sequence of LaAXE, the three-dimensional model structure was constructed via the Phyre^2^ 3D-modelling server [30]. LaAXE shares the highest structural similarity (41%) with the *Thermoanaerobacterium* AXE (PDB ID: 3FCY). The monomeric model of LaAXE possesses the α/β-hydrolase fold consisting of the central eight paralleled β-sheets flanked by α-helices, which is highly conserved in the known structures of AXEs CE7 from *Thermotoga maritima* (5FDF), *Thermoanaerobacterium* sp. (3FCY), *B. pumilus* (2XLB), and *B. subtilis* (1L7A). These AXEs share very similar quaternary structures of homo-hexameric oligomer consisting of a trimer of dimers [11, 31].

According to the primary and tertiary structure analyses, LaAXE and BhAXE could be classified as the new members of AXE CE family 7. Even though the activity towards 7-ACA was not so high, the first AXE CE7 with broad substrate specificity was functionally characterized from *Lb. antri*, a strain of lactic acid bacteria. It possesses the considerable deacetylating activities against beechwood xylan and its hydrolysates, which can promote the successive actions of β-xylanase. When the modes of action of LaAXE are understood in detail, it will be one of the better candidates for the enzymatic production of antibiotics and prebiotic carbohydrate materials.

## Figures and Tables

**Table 1:** Specific activities of AXEs on various acetylated substrates.

Substrates^a^	Specific activity (U/mg)	

	LaAXE	BhAXE
GPAc	499.5 ± 6.3 (100.0%)	501.0 ± 20.0 (100.0%)
4-MUAc	48.7 ± 0.7 (9.7%)	293.0 ± 0.0 (58.5%)
*p*-NPAc	202.9 ± 6.6 (40.6%)	134.6 ± 2.4 (26.9%)
7-ACA	20.7 ± 0.6 (4.1%)	165.0 ± 0.7 (32.9%)

^a^GPAc, β-D-glucose pentaacetate; 4-MUAc, 4-methyl umbelliferyl acetate; *p*-NPAc, *p*-nitrophenyl acetate; 7-ACA, 7-aminocephalosporanic acid
